# Supplementary material for: Constructing the hierarchy of predictive auditory sequences in the marmoset brain
Source: eLife. 2022 Feb 17;11:e74653. doi: 10.7554/eLife.74653 (PMC8893719; doi:10.7554/eLife.74653)
Supplement: Supplementary file 1. — (a): The peak fMRI activations of brain regions for 1st-level novelty. The image results were zoomed into human brain size for visualization and using MNI coordinates. IC, inferior colliculus; MG, medial geniculate nucleus; AuA1, primary auditory area; AuR, rostral auditory area; AuCM, caudomedial auditory area. L, left; R, right. (b): The peak fMRI activations of brain regions for 2nd-level novelty by comparing xx|xY with xY|xY. The image results were zoomed into human brain size for visualization and using MNI coordinates. AuAL, anterolateral auditory area; STR, superior temporal rostral area. (c): The peak fMRI activations of brain regions for both 1st- and 2nd-level novelty by comparing xY|xx with xx|xx. The image results were zoomed into human brain size for visualization and using MNI coordinates. AuRT, rostrotemporal auditory area; AuRTM, rostrotemporal medial auditory area; AuRPB, rostral parabelt auditory area; PE, parietal area. (d): The peak fMRI activations of brain regions for omission of the 5th tone in xx task by comparing x_|xx with xx|xx. The image results were zoomed into human brain size for visualization and using MNI coordinates. TE3, inferior temporal cortex. (e): The peak fMRI activations of brain regions for omission of the 5th tone in xY task by comparing x_|xY with xY|xY. The image results were zoomed into human brain size for visualization and using MNI coordinates. AuML, middle lateral auditory area. (f): The peak fMRI activations of brain regions for difference between local deviant and local standard omissions by comparing x_|xY with x_|xx. The image results were zoomed into human brain size for visualization and using MNI coordinates. [file elife-74653-supp1.docx]

**Supplementary File 1:** Details of fMRI results for brain regions of interest.

| **Supplementary File 1a:** The peak fMRI activations of brain regions for 1^st^-level novelty. The image results were zoomed into human brain size for visualization and using MNI coordinates. IC, inferior colliculus; MG, medial geniculate nucleus; AuA1, primary auditory area; AuR, rostral auditory area; AuCM, caudomedial auditory area. L, left; R, right. | | | | | | |
| --- | --- | --- | --- | --- | --- | --- |
| Brain region | Laterality | t-value | Cluster size | MNI coordinates | | |
|  |  |  |  | x | y | z |
| IC | R | 2.58 | 27 | 15 | -65 | 1 |
|  | L | 2.27 | 11 | -9 | -64 | 4 |
| MG | R | 3.17 | 9 | 33 | -37 | -6 |
|  | L | 2.71 | 6 | -23 | -37 | -14 |
| AuA1 | R | 3.1 | 376 | 46 | -26 | 22 |
|  | L | 3.1 | 579 | -48 | -28 | 33 |
| AuR | R | 3.41 | 204 | 54 | 1 | 9 |
| AuCM | R | 2.74 | 14 | 52 | -16 | 3 |
| A23a | R | 2.67 | 26 | 6 | -26 | 36 |
| A23b | L | 2.13 | 10 | -3 | -31 | 39 |
| A24a | R | 3.32 | 23 | 7 | 14 | 18 |
| A24b | L | 2.08 | 18 | 0 | 14 | 25 |
| A8b | R | 3 | 128 | 3 | 28 | 34 |
| A10 | L | 2.46 | 15 | 0 | 50 | 9 |

| **Supplementary File 1b:** The peak fMRI activations of brain regions for 2^nd^-level novelty by comparing xx\|xY with xY\|xY. The image results were zoomed into human brain size for visualization and using MNI coordinates. AuAL, anterolateral auditory area; STR, superior temporal rostral area. | | | | | | |
| --- | --- | --- | --- | --- | --- | --- |
| Brain region | Laterality | t-value | Cluster size | MNI coordinates | | |
|  |  |  |  | x | y | z |
| AuAL | R | 3.58 | 28 | 61 | -8 | -6 |
|  | L | 4.19 | 78 | -60 | -2 | -11 |
| AuCM | R | 5.72 | 97 | 45 | -13 | 10 |
|  | L | 4.6 | 22 | -54 | -31 | 10 |
| STR | R | 4.72 | 120 | 54 | 7 | -20 |
| A32 | L | 4.5 | 265 | -8 | 34 | 9 |
| A23a | R | 4.45 | 32 | 10 | -5 | 22 |
|  | L | 4.73 | 123 | -8 | -20 | 28 |
| A3b | R | 5.65 | 271 | 21 | -22 | 39 |
| A4ab | L | 4.5 | 46 | -30 | -5 | 36 |
| A8aV | R | 5.29 | 162 | 19 | 41 | 16 |
|  | L | 4.82 | 142 | -27 | 25 | 16 |
| A13 | L | 4.91 | 237 | -11 | 26 | -6 |

| **Supplementary File 1c:** The peak fMRI activations of brain regions for both 1^st^- and 2^nd^-level novelty by comparing xY\|xx with xx\|xx. The image results were zoomed into human brain size for visualization and using MNI coordinates. AuRT, rostrotemporal auditory area; AuRTM, rostrotemporal medial auditory area; AuRPB, rostral parabelt auditory area; PE, parietal area. | | | | | | |
| --- | --- | --- | --- | --- | --- | --- |
| Brain region | Laterality | t-value | Cluster size | MNI coordinates | | |
|  |  |  |  | x | y | z |
| MG | R | 5.12 | 44 | 25 | -40 | -11 |
|  | L | 5.64 | 80 | -29 | -38 | -6 |
| AuA1 | L | 6.37 | 1597 | -63 | -14 | 15 |
| AuRT | R | 6.99 | 1258 | 57 | -2 | 0 |
|  | L | 4.3 | 25 | -63 | 4 | -3 |
| AuRTM | L | 5.33 | 118 | -54 | -2 | -8 |
| AuRPB | R | 4.14 | 24 | 60 | -7 | -18 |
| A31 | L | 4.82 | 88 | -8 | -31 | 51 |
| A23b | R | 4 | 16 | 6 | -23 | 37 |
| PE | L | 5.17 | 580 | -17 | -23 | 43 |
| A3a | L | 4.4 | 96 | -36 | -5 | 39 |
| A8b | R | 3.77 | 26 | 9 | 26 | 37 |
| A10 | L | 3.81 | 34 | -12 | 53 | 13 |

| **Supplementary File 1d:** The peak fMRI activations of brain regions for omission of the 5^th^ tone in xx task by comparing x_\|xx with xx\|xx. The image results were zoomed into human brain size for visualization and using MNI coordinates. TE3, inferior temporal cortex. | | | | | | |
| --- | --- | --- | --- | --- | --- | --- |
| Brain region | Laterality | t-value | Cluster size | MNI coordinates | | |
|  |  |  |  | x | y | z |
| TE3 | R | 4.09 | 30 | 63 | -28 | -24 |
|  | L | 3.88 | 23 | -65 | -28 | -24 |
| A8aV | R | 3.99 | 46 | 36 | 35 | 21 |
| A47L | L | 3.83 | 13 | -45 | 28 | 1 |
| A23a | R | 3.77 | 17 | 3 | -10 | 27 |
|  | L | 5.12 | 155 | -11 | -16 | 25 |
| A23b | R | 4.23 | 45 | 9 | -20 | 39 |
|  | L | 4.98 | 92 | -5 | -19 | 45 |
| A30 | R | 3.9 | 69 | 9 | -26 | 22 |

| **Supplementary File 1e:** The peak fMRI activations of brain regions for omission of the 5^th^ tone in xY task by comparing x_\|xY with xY\|xY. The image results were zoomed into human brain size for visualization and using MNI coordinates. AuML, middle lateral auditory area. | | | | | | |
| --- | --- | --- | --- | --- | --- | --- |
| Brain region | Laterality | t-value | Cluster size | MNI coordinates | | |
|  |  |  |  | x | y | z |
| AuML | R | 4.06 | 19 | 64 | -17 | 0 |
|  | L | 4.84 | 41 | -60 | -26 | 10 |
| A13M | R | 5.26 | 68 | 16 | 28 | -2 |
|  | L | 4.9 | 62 | -23 | 25 | -5 |
| A47M | R | 4.61 | 43 | 30 | 29 | 2 |
| A24d | R | 4.88 | 71 | 15 | -2 | 36 |
| A32 | L | 4.15 | 30 | -11 | 37 | 9 |

| **Supplementary File 1f:** The peak fMRI activations of brain regions for difference between local deviant and local standard omissions by comparing x_\|xY with x_\|xx. The image results were zoomed into human brain size for visualization and using MNI coordinates. | | | | | | |
| --- | --- | --- | --- | --- | --- | --- |
| Brain region | Laterality | t-value | Cluster size | MNI coordinates | | |
|  |  |  |  | x | y | z |
| AuA1 | L | 4.9 | 81 | -60 | -25 | 12 |
| AuAL | R | 4.47 | 21 | 67 | -7 | -5 |
| A8b | R | 4.19 | 14 | 9 | 34 | 24 |
|  | L | 4.06 | 16 | -5 | 23 | 45 |
| A24c | R | 3.84 | 15 | 12 | 29 | 18 |
| A9 | L | 4.02 | 37 | -6 | 53 | 18 |
| A10 | L | 4.21 | 27 | -23 | 49 | 22 |
