# Supplementary material for: Constructing the hierarchy of predictive auditory sequences in the marmoset brain
Source: eLife. 2022 Feb 17;11:e74653. doi: 10.7554/eLife.74653 (PMC8893719; doi:10.7554/eLife.74653)
Supplement: Transparent reporting form [file elife-74653-transrepform1.docx]

***eLife’s* transparent reporting form**

We encourage authors to provide detailed information *within their submission* to facilitate the interpretation and replication of experiments. Authors can upload supporting documentation to indicate the use of appropriate reporting guidelines for health-related research (see [EQUATOR Network](http://www.equator-network.org/%20)), life science research (see the [BioSharing Information Resource](https://biosharing.org/)), or the [ARRIVE guidelines](http://www.plosbiology.org/article/info:doi/10.1371/journal.pbio.1000412) for reporting work involving animal research. Where applicable, authors should refer to any relevant reporting standards documents in this form.

If you have any questions, please consult our Journal Policies and/or contact us: [editorial@elifesciences.org](mailto:editorial@elifesciences.org).

**Sample-size estimation**

- You should state whether an appropriate sample size was computed when the study was being designed
- You should state the statistical method of sample size computation and any required assumptions
- If no explicit power analysis was used, you should describe how you decided what sample (replicate) size (number) to use

Please outline where this information can be found within the submission (e.g., sections or figure legends), or explain why this information doesn’t apply to your submission:

According to previous monkey studies, we used 3 marmoset monkeys for fMRI experiments and 2 marmoset monkeys for ECoG experiments. The age and gender of marmosets used in the fMRI and ECoG experiments were matched. The marmoset information can be found in the sections “fMRI” and “ECoG implantation and recording”.

**Replicates**

- You should report how often each experiment was performed
- You should include a definition of biological versus technical replication
- The data obtained should be provided and sufficient information should be provided to indicate the number of independent biological and/or technical replicates
- If you encountered any outliers, you should describe how these were handled
- Criteria for exclusion/inclusion of data should be clearly stated
- High-throughput sequence data should be uploaded before submission, with a private link for reviewers provided (these are available from both GEO and ArrayExpress)

Please outline where this information can be found within the submission (e.g., sections or figure legends), or explain why this information doesn’t apply to your submission:

In fMRI experiments, we collected 20-25 sessions of data for each marmoset, each session consisted of 3-4 runs of each task, which depended on the marmosets’ performance on that day. In ECoG experiments, we collected 20-25 sessions of data for each marmoset, each session consisted of 1-2 runs of each task. The replicates information and criteria for exclusion/inclusion of data can be found in the sections of “Auditory paradigm”, “fMRI data analysis” and “ECoG data analysis”.

**Statistical reporting**

- Statistical analysis methods should be described and justified
- Raw data should be presented in figures whenever informative to do so (typically when N per group is less than 10)
- For each experiment, you should identify the statistical tests used, exact values of N, definitions of center, methods of multiple test correction, and dispersion and precision measures (e.g., mean, median, SD, SEM, confidence intervals; and, for the major substantive results, a measure of effect size (e.g., Pearson's r, Cohen's d)
- Report exact p-values wherever possible alongside the summary statistics and 95% confidence intervals. These should be reported for all key questions and not only when the p-value is less than 0.05.

Please outline where this information can be found within the submission (e.g., sections or figure legends), or explain why this information doesn’t apply to your submission:

For fMRI data, a within-subject analysis of variance (one-way ANOVA) was used for regular and omission local-global experiments, respectively. The statistical information can be found in the section of ” fMRI data analysis”.

For ECoG data, a non-parametric permutation test was used for comparison. The statistical information can be found in the section of ” ECoG data analysis”. The dispersion and precision measures of γ- and β-band activities were described in the section of ECoG part of “Results”.

The details of the analysis of functional correlation were included in the section of ”ECoG data analysis”. The statistical results can be found in Figs. 3C and D, Figs. 5C and D.

(For large datasets, or papers with a very large number of statistical tests, you may upload a single table file with tests, Ns, etc., with reference to sections in the manuscript.)

**Group allocation**

- Indicate how samples were allocated into experimental groups (in the case of clinical studies, please specify allocation to treatment method); if randomization was used, please also state if restricted randomization was applied
- Indicate if masking was used during group allocation, data collection and/or data analysis

Please outline where this information can be found within the submission (e.g., sections or figure legends), or explain why this information doesn’t apply to your submission:

In the fMRI/ECoG experiments, the marmosets underwent the same auditory paradigm. The xx task using xY|xx as deviant, the xx task using omission as deviant, the xY task using xx|xY as deviant, the xY task using omission as deviant were presented pseudo randomly.

**Additional data files (“source data”)**

- We encourage you to upload relevant additional data files, such as numerical data that are represented as a graph in a figure, or as a summary table
- Where provided, these should be in the most useful format, and they can be uploaded as “Source data” files linked to a main figure or table
- Include model definition files including the full list of parameters used
- Include code used for data analysis (e.g., R, MatLab)
- Avoid stating that data files are “available upon request”

Please indicate the figures or tables for which source data files have been provided:

The toolbox we used in the experiment and analyses, including Matlab, Psychophysics Toolbox, SPM12, FieldTrip and N-way, were described in the section of “fMRI”, ” fMRI data analysis” and ” ECoG data analysis”.
